# Bridging the Gap between Philosophers of Mind and Brain Researchers: The Example of Addiction **

**DOI:** 10.4103/0973-1229.77435

**Published:** 2011

**Authors:** Christian Perring

**Affiliations:** * *Department of Philosophy, Dowling College, 150 Idle Hour Blvd, Oakdale, NY 11769, USA*; ** *Revised and peer reviewed version of a paper for an International Seminar on Mind, Brain, and Consciousness, Thane College Campus, Thane, India, January 13-15, 2010.*

**Keywords:** *Addiction*, *Action theory*, *Compulsion*, *Philosophy*, *Selfcontrol*

## Abstract

Philosophers and psychologists have long tried to understand people’s irrational behaviour through concepts such as weakness of will, compulsion and addiction. The scientific basis of the project has been greatly enhanced by advances in cognitive psychology and neuroscience. However, some philosophers have also been critical of the more general conclusions drawn by the scientists. This is especially true when scientific researchers start making claims that go to philosophical issues, such as free will and responsibility. Conversely, some scientists have been critical of philosophical approaches for not understanding the results of recent research. I examined some of the recent history of scientific claims about addiction, and the rise of the claims from scientists to have shown that addiction is a brain disease and that addictive behaviour is compulsive. Given the well-confirmed evidence that addicts can modulate their behaviour in response to rewards, punishments and context, it is clear that according to normal definitions of compulsivity the behaviour of addicts is not typically compulsive, suggesting that neuroscientists are making an error in their interpretation of data. Since philosophers have expertise in making distinctions between different kinds of action and categorising them as free, weak-willed and compulsive, we will achieve a better interpretation of the neuroscience of addiction when taking this philosophical work into account. Conversely, given the status of science in the modern world, philosophers have to grapple with the latest neuroscientific discoveries and show the compatibility of their philosophical theories with the data for their approaches to maintain credibility.

## Introduction

The concept of addiction as a disease came to be taken seriously in the USA and in England at the start of the nineteenth century (Levine, 1978), but it has had a disputed status from its inception and there continues to be debate over whether it is really a disease to the present day (Heyman, 2009; Valverde, 1998). In recent years, some neuroscientists have argued that neuroscience can demonstrate the reality of the disease of addiction (Leshner, 1997; Volker and Fowler, 2000). Yet, some philosophers have argued that it would take a great deal for neuroscience to be able to demonstrate that any action is truly compulsive, rather than simply giving an account of the causal pathways leading to addictive behaviour (Stephens and Graham, 2009). We would benefit from further discussion of how we can achieve a productive debate between neuroscientists and philosophy, so as to move the debate along and establish a better foundation for public policy. I will argue that when neuroscience addresses concepts such as freedom, compulsion and disease, it benefits from philosophical understanding, and conversely, philosophers who address similar concepts in psychiatry benefit from understanding the data provided by neuroscience.

The concept of disease, especially of psychiatric disease, takes a great deal of unpacking. It is doubtful that we can achieve a precise and uncontroversial definition. However, for our purposes with regards to the issue of addiction, we can focus on its connection with involuntary or non-autonomous action. If addiction is truly compulsive and addictive action is not voluntary, then addiction has a much stronger claim to be a disease. Most of the best-known criticisms of the disease concept (e.g., Fingarette, 1988; Peele, 1999) have argued that addiction is not a disease because addictive behaviour is voluntary. In order to determine whether addictive action is voluntary or not, we need to specify what we mean.

The debate between philosophy and neuroscience has been dishearteningly confused when it comes to personal freedom. A paradigm of this has been Libet’s (1985) notorious claim to have shown that human action is not under conscious control, which has been used by others (e.g., Wegner, 2002) to support the claim that free will is an illusion. These analyses have now been largely shown to be problematic (O’Connor, 2005; Mele, 2006; Mele, 2009), but one wonders whether it might not have been possible to forestall the erroneous interpretations of the original experiments with better dialogue between neuroscientists and philosophers early on in the process. This ambition is complicated by the fact that there is still debate within philosophy about the nature of voluntary action and personal autonomy, and it is easy to understand the temptation of arguing for a shocking conclusion that we have no free will, a view which is often appealing to those who take a scientific view of human behaviour. Nevertheless, my claim here is that we will make more progress by increasing the dialogue between philosophers and neuroscientists, and that we need to make clearer the conceptual work in claims of reduced autonomy. With this, we can work toward a more sophisticated understanding of addiction that avoids simplistic views of complete freedom or complete lack of freedom. This will better enable us to understand the difference in responsibility between addicts and non-addicts.

## Addiction in Neuroscience

Let us consider the argument in the review article of Volkow and Fowler (2000). They pointed out that the orbitofrontal cortex is an area of the brain that could integrate information from various limbic areas of the brain, which could modulate the response of those areas to drugs. They made many connected observations about the orbitofrontal cortex, including the following. In dopamine transporter knockout mice, self-administration of cocaine results in activation of the area. In human subjects, the area has been associated with reinforced behaviour and conditioned responses. Pathology in the orbitofrontal cortex and striatum have been reported in people with OCD, and in the same parts, increases in metabolic activity are found in people with obsessions, compulsions, impulsivity and Tourette’s syndrome. The authors mention a case in which a person with a vascular lesion of the orbitofrontal cortex compulsively borrowed cars illegally, leading to multiple incarcerations. Hyperactivity of the orbitofrontal cortex appears to be associated with reports of the cravings of cocaine addicts. “Imaging studies have provided evidence of abnormalities in the striatum, thalamus and orbitofrontal cortex in cocaine abusers” (p322) and similarly in alcoholics. After setting out a wide range of other similar pieces of evidence, the authors conclude that “we postulate that repeated exposure to drugs of abuse disrupts the function of the striato-thalamo-orbitofrontal circuit. As a consequence of this dysfunction a conditioned response occurs when the addicted subject is exposed to the drug and/or drug-related stimuli that activates this circuit and results in the intense drive to get the drug (consciously perceived as a craving) and compulsive self-administration of the drug (consciously perceived as a loss of control).” (p323)

The logic of the paper is unclear. In particular, the paper is full of associations between ideas and apparent implications, and the conclusion is a hypothesis. The authors do not specifically say whether the considerations they list in the main body of the paper are evidence for the postulate. However, the argument seems to be spelled out most clearly in the abstract: “Because the orbitofrontal cortex is involved with drive and with compulsive repetitive behaviours, its abnormal activation in the addicted subject could explain why compulsive drug self-administration occurs even with tolerance to the pleasurable drug effects and in the presence of adverse reactions.” (p318). So, essentially the argument is that because a kind of brain activation distinctively associated with Tourette’s, OCD and impulsivity is also associated with addiction, there is reason to think that addiction is compulsive.

As a hypothesis, it is certainly worth pursuing. However, the paper never spells out what it means by compulsivity. The closest it comes is in making a distinction between pleasure-seeking activity and compulsive action. The idea is that people will sometimes pursue a course of action even when it may result in unpleasant outcomes. Yet, it by no means follows that such action is unfree or compulsive. It is relatively uncontroversial that people can be irrational and free and many philosophers have argued that weak-willed action does not need to be compulsive (Mele, 1987). That is to say, people choose to act against their own better judgment when they could have acted otherwise. So, Volkow and Fowler need further argument that the phenomena they are discussing are truly compulsive.

## Neuroscience, Psychology and Philosophy

Some might defend Volkow and Fowler by pointing out that it is unfair to focus on their paper without including discussion of the supporting structure of knowledge from neuroscience and its considerable investigation of action and pathologies of action. It is certainly true that we need to take a larger view in addressing the contribution of neuroscience in understanding the nature of addiction. Nevertheless, it is reasonable to address a much-cited paper in the terms that it lays out its own argument. The criticisms made above still apply.

In another move, some might defend Volkow and Fowler by arguing that they are not in a position to review all the literature and although it is true that they do not address alternative views regarding addiction, they do not need to do so. They know who their opponents are, with a large literature setting out the debate, and so the arguments do not need to be set out again in a review paper, so the defence might go. However, this will not do. Apart from the fact that the Volkow and Fowler review paper makes no reference to the debate over the disease status of addiction, it is obviously not a debate that has been settled because the whole point of their paper is to make a case for their side. So, to make a convincing case regarding a contested issue, it is necessary to address both sides and to examine the case against the disease status of addiction. If it is not possible in such a context to make the full argument, then the authors should explicitly restrict the status of their conclusion.

To focus on one paper is far from evaluating the whole literature in neuroscience on the nature of addiction. Yet, the argument so far does at least force us to focus on the difficulty of integrating the different theories of mind and pathology, a problem recently highlighted by Ghaemi (2009) and West (2006). We do not have a clear model for how to match together different psychological theories at different levels of description, both those that are empirically testable and also those from folk psychology and ordinary language. This is especially clear in the case of substance dependence.

## Evidence that Addiction is Voluntary

One of the primary empirical studies that suggest that addiction has a voluntary component is the study by Lee Robins in the 1970s of the heroin addiction of US soldiers in Vietnam and their rapid recovery rates on returning to the USA. The study found that in the first year after return, only 5% of those who had been addicted remained so (Robins, 1993, p. 1045). Heyman (2009) points out that offering addicts incentives to change their behaviour is often successful. For example, Higgins has done work with the behavioural treatment of addiction. His group offered cocaine addicts cash incentives to refrain from using the drug, and were also given education about drug use and its consequences (Higgins, 1991). The results were that the behavioural treatment led to significantly longer abstinence from drugs than 12-Step treatment. Heyman cites other studies showing that addicts do tend to reduce or end their substance use when they face negative repercussions.

Thus, we can conclude that there is countervailing evidence to the neuroscientific studies, and we should remember that those studies did not provide conclusive arguments, even according to their own authors. What is puzzling is how Volkow and her colleagues can even countenance the hypothesis that addiction is compulsive, given the weighty set of evidence that addicts can modulate their behaviour according to circumstances. It might be possible to preserve the claim that addiction involves compulsive action if a more precise and narrow definition of addiction is used, restricting it to the most serious cases, but this is not the strategy taken by these neuroscientists.

This leaves the neuroscientific defence of a disease model of addiction from Volkow and Fowler in a difficult position. Although it has demonstrated some parallels between addiction and other mental disorders such as OCD and Tourette’s, these do not provide sufficient reason to conclude that addictive behaviour is involuntary. Even if there were a very strong parallel between addiction and these other mental disorders, it would not automatically follow that addiction was involuntary, because the involuntary nature of behaviour associated with OCD and Tourette’s is at least up for debate and further investigation. For example, Oliver Sacks’ well-known discussion of Tourette’s in his essay “A Surgeon’s Life” (1995) says that people with this condition are able to delay their ticking behaviour when they need to, at least for a certain amount of time. One finds similar claims with regard to narrative accounts of OCD, in which sufferers of the condition say that they are able to refrain from their compulsive behaviour for some time, but if they do so, then they need to immerse themselves in their rituals when they do give in to them. This suggests that people have an ability to resist their compulsions, but that their reserves of resistive energy are limited, this is an idea strongly reminiscent of the phenomenon of ego-depletion investigated by social psychologist Roy Baumeister (2007). If we take this idea seriously, then we might be able to revive the compulsiveness of addiction in a more sophisticated manner (see Levy, 2007, for an attempt to do this).

## A Balance between Neuroscience and Philosophy

The nature of compulsion, whether in OCD, Tourette’s, impulsive behaviour or addiction, is not well understood either by philosophy, social psychology or cognitive neuroscience. Voluntary action is a complex phenomenon, and the concept of voluntariness is not precise in ordinary language. To be capable of serious scientific investigation, it needs to be carefully defined. The concept of involuntary action is especially difficult to grasp, and the above discussion suggests that we might do better to conceptualise the phenomenon differently. For example, we might use the concept of degrees of freedom and reductions in autonomy. It is clear that there is no straightforward dichotomy between voluntary and involuntary action, and so, we need to employ a conceptual apparatus adequate to the task of description of the phenomena under investigation.

This brings me back to my main claim in this paper. Philosophers have been investigating the concept of action, both voluntary and involuntary, since the start of the discipline. They have already developed theories and vocabularies which address the complexity of the phenomenology of compromised and reduced autonomy. Neuroscientific approaches to addiction and other forms of psychopathology will be improved by a more sophisticated and philosophically informed set of grounding concepts. Then, it will be possible to better understand the evidence from neuroscience and to employ it in public policy.

It is important to see that this is not a one-way street. The evidence from neuroscience is also important for our philosophical understanding of persons. The fact that there are similar patterns of brain activation in addiction and other forms of psychopathology involving bizarre, inappropriate and self-defeating action helps us to conceptualise it better. Using the phenomenology given in self-reports is notoriously unreliable, as a guide to the nature of action and philosophers need to embrace the better quantifiable results from brain scans as a new way of grounding our understanding, just as they need to explore work in social psychology. Exactly how this information can be integrated into our philosophical articulations of our self-conception is not yet clear, and this is an exciting new challenge for philosophy. Ideally, we would do better to reduce the gap between the disciplines of philosophy and neuroscience and work toward a synthesis of approaches. In order to achieve this, we need to be able to communicate and collaborate on the interpretation of experimental results.

## Concluding Remarks [see also Figure 1]

**Figure 1:**
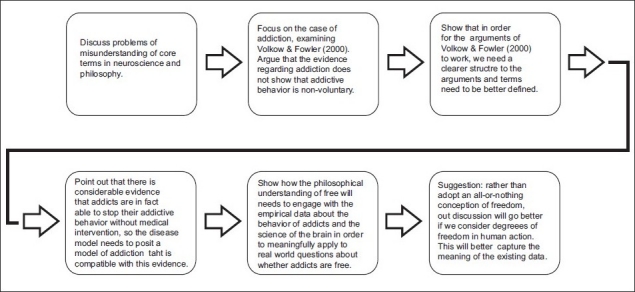
Flowchart of paper

In working toward the best possible understanding of people with substance abuse problems, both philosophy and neuroscience have an important role to play in considering to what extent they have the ability to control themselves and act otherwise. We can make comparisons between these problems, normal everyday behaviour where we believe that people do have self-control and mental disorders where we are more confident that the sufferer is unable to control themselves, and both neuroscience and phenomenology will play a role. By integrating a variety of perspectives, we will be in a position to decide whether these problems should qualify as a disease, with all the social implications that follow from this judgment.

## Take home message

When addressing the nature of addiction, neuroscience and philosophy need each other. Neuroscience runs the risk of undermining itself by making simplistic claims about compulsion that do not mesh with well-established results about addict’s abilities to do otherwise. Philosophy runs the risk of being empirically out of touch if it has no connection with the latest scientific findings. Yet, if the two disciplines collaborate in interpretation, they have the potential of being mutually enriching and thus achieving a sophisticated and helpful understanding of this perplexing phenomenon.

## Questions That This Paper Raises

1. Can neuroscience tell us what is going on in a person’s consciousness?
2. When does a similarity in brain activation between two conditions of a person warrant a conclusion that those conditions are fundamentally similar?
3. What sorts of problems with brain function entitle us to conclude that the problems should count as a brain disease?
4. When trying to understand whether a person has self-control, how do we weigh the importance of phenomenology relative to that of brain science?
5. Can philosophy provide a unifying and grounding conceptual scheme with which we can formulate our understanding of self-control that will make a variety of different psychological theories commensurable?

## About the Author

Christian Perring is Professor of Philosophy at Dowling College, New York, USA. He is Department Chair, and he teaches a wide range of subjects, including philosophy of psychiatry, health care ethics and the philosophy of sex and love. He is editor of Metapsychology Online Reviews [http://metapsychology.mentalhelp.net/] and he is on the Executive Council of the Association for the Advancement of Philosophy and Psychiatry. He has published articles on the philosophy of psychiatry in a variety of journals and has also edited books. He is working on a book on the moral responsibility of the actions of people with mental illnesses.
